# An Efficient Data Structure and Algorithm for Long-Match Query in Run-Length Compressed BWT

**DOI:** 10.4230/LIPIcs.WABI.2025.17

**Published:** 2025-08-15

**Authors:** Ahsan Sanaullah, Degui Zhi, Shaojie Zhang

**Affiliations:** Department of Computer Science, University of Central Florida, Orlando, FL, USA; McWilliams School of Biomedical Informatics, University of Texas Health Science Center at Houston, TX, USA; Department of Computer Science, University of Central Florida, Orlando, FL, USA

**Keywords:** BWT, LEM, Long LEM, MEM, Run Length Compressed BWT, Move Data Structure, Pangenome, Theory of computation → Pattern matching, Theory of computation → Data compression

## Abstract

String matching problems in bioinformatics are typically for finding exact substring matches between a query and a reference text. Previous formulations often focus on maximum exact matches (MEMs). However, multiple occurrences of substrings of the query in the text that are long enough but not maximal may not be captured by MEMs. Such long matches can be informative, especially when the text is a collection of similar sequences such as genomes. In this paper, we describe a new type of match between a pattern and a text that aren’t necessarily maximal in the query, but still contain useful matching information: locally maximal exact matches (LEMs). There are usually a large amount of LEMs, so we only consider those above some length threshold ℒ. These are referred to as long LEMs. The purpose of long LEMs is to capture substring matches between a query and a text that are not necessarily maximal in the pattern but still long enough to be important. Therefore efficient long LEMs finding algorithms are desired for these datasets. However, these datasets are too large to query on traditional string indexes. Fortunately, these datasets are very repetitive. Recently, compressed string indexes that take advantage of the redundancy in the data but retain efficient querying capability have been proposed as a solution. We therefore give an efficient algorithm for computing all the long LEMs of a query and a text in a BWT runs compressed string index. We describe an O(m+occ) expected time algorithm that relies on an O(r) words space string index for outputting all long LEMs of a pattern with respect to a text given the matching statistics of the pattern with respect to the text. Here m is the length of the query, occ is the number of long LEMs outputted, and r is the number of runs in the BWT of the text. The O(r) space string index we describe relies on an adaptation of the move data structure by Nishimoto and Tabei. We are able to support LCP[i] queries in constant time given SA[i]. In other words, we answer PLCP[i] queries in constant time. These PLCP queries enable the efficient long LEM query. Long LEMs may provide useful similarity information between a pattern and a text that MEMs may ignore. This information is particularly useful in pangenome and biobank scale haplotype panel contexts.

## 1 Introduction

Bioinformatics sequence data is often large and very repetitive. Furthermore, efficient matching queries on the data are frequently needed for many biological analyses. Therefore, bioinformatics problems have incentivized and profited from the development of efficient string indexes. The Burrows-Wheeler transform (BWT) has thus been used in bioinformatics algorithms. The BWT is a permutation of a text that has found wide use in string indexing and data compression [11]. Position i in the BWT of the text is essentially the character preceding the i-th lexicographically smallest suffix of the text. Due to this lexicographic sorting, adjacent characters in the BWT correspond to the characters preceding highly locally similar suffixes of the text. Therefore, the BWT of highly repetitive texts tends to have large runs of one character, with an overall small number of runs. The BWT of highly repetitive texts therefore compresses well. In fact, the number of runs in BWT, r, is sometimes used as a measure of the repetitiveness of a string [35]. Finally, given only the BWT of a text, the text can be reconstructed in linear time [11] and the BWT of a text can be constructed in linear time by construction of the suffix array [22]. The BWT ordering also allows efficient string indexes. In other words, given a pattern, find all occurrences of the pattern within the text. String indexes have been shown that output all occurrences of a pattern (a locate query) in space linear to the product of the length of the text and the size of the alphabet and time linear to the sum of the length of the pattern and the number of occurrences [18].

Compressed string indexes have also been shown [3, 18, 32]. These indexes output all occurrences of a pattern in space sublinear to the size of the text. Although the time complexity of locating these occurrences is not linear in the length of the pattern and the number of occurrences, they are typically independent of the length of the text barring logarithmic factors and close to linear in the length of the pattern and number of occurrences. In particular for highly repetitive texts, the space of the index can be much smaller than the space of the text. Notably, recent compressed string indexes have achieved space linear to the number of runs in the BWT (r) [19,36]. The r-index by Gagie et al. was the first compressed string index offering close to linear time locate queries in O(r) space [19]. Nishimoto and Tabei recently improved on this result with their OptBWTR, which achieves linear time locate queries for texts with alphabets of size polylogarithmic in the length of the text. OptBWTR relies on the move data structure, which was introduced in the same paper [36].

Compressed string indexes have been fruitfully applied to the growing collection of bioinformatics data. Over the past two decades, large collections of genomics data have grown increasingly larger in size. For example, the UK Biobank has whole genome sequencing data of roughly one million haplotypes [29], and the All of Us program has released whole genome sequencing data of half a million haplotypes [7]. Furthermore, recent arguments have been made that a human reference pangenome should be used instead of a singular human reference genome to avoid reference bias in downstream analyses [33, 43, 46]. The Human Pangenome Reference Consortium has released a draft human pangenome reference of more than two hundred high quality phased diploid assemblies and is planning to release over three hundred and fifty in the final release [12,30]. The UK Biobank whole genome sequencing data has 1.5 billion variants, the All of Us whole genome sequencing data has 1 billion variants, and the typical diploid assembly in the draft human pangenome has 6 billion bases. Therefore, these datasets have 1,500 trillion, 250 trillion, and 1.2 trillion characters each respectively. However, while very large, these datasets are very repetitive. Furthermore, queries on these datasets are frequently needed for biological applications including read mapping [24, 27], read alignment [28], read classification for metagenomes [1, 15, 45] or pangenomes [10]. Many general purpose compressed string indexes have also been implemented for exact pattern matching and matching substrings of the pattern [16, 26, 38, 49]. These indexes may compute the maximal exact matches (MEMs) of the pattern with respect to the text. MEMs are matches between the pattern and the text that cannot be extended in the pattern.

While MEMs typically refer to matches that are maximal in the pattern, matches that are simultaneously maximal in the pattern and the text may sometimes be desired. Notably, in two data structures related to the BWT, algorithms for outputting matches that are simultaneously maximal in the pattern and the text have already been developed. These data structures are the positional Burrows-Wheeler transform (PBWT) and the graph Burrows-Wheeler transform (GBWT) [17, 44]. In the PBWT and GBWT, matches that cannot be extended in the pattern are referred to as set maximal matches, and matches that cannot be simultaneously extended in the pattern are referred to as locally maximal matches. Locally maximal matches that are longer than some length threshold ℒ are referred to as ℒ-long matches, or long matches for short. Algorithms for outputting set maximal matches and long matches have been published in the PBWT in uncompressed [17, 34, 40] and compressed space [8, 13, 42, 48]. Algorithms for outputting these matches have also been published for the GBWT in compressed space [39].

In this paper, we use these concepts in the traditional pattern and text context, and name matches that cannot be simultaneously extended in the pattern and the text locally maximal exact matches (LEMs). LEMs that are longer than ℒ are long LEMs. The distinction between matches that do not extend in the pattern and matches that do not extend simultaneously in the pattern and the text has been made before. Notably, in ropebwt3 MEMs refers to LEMs of our paper and super maximal exact matches (SMEMs) refers to MEMs of our paper [26]. The term SMEM has been used in place of MEM in a few papers to avoid the confusion in terminology [8, 13, 15, 26], however MEM is still the most common term by far for matches that cannot be extended in the pattern. The authors are not aware of any published algorithms for the computation of LEMs or long LEMs.

In this work, we describe an algorithm for outputting all long LEMs of a pattern with respect to a text in O(m+occ) expected time given the matching statistics of the pattern with respect to the text, where m is the length of the pattern and occ is the number of long LEMs it has with respect to the text. In order to do so, we modify the OptBWTR data structure of Nishimoto and Tabei to also compute LCP[i] given SA[i] (i.e. compute PLCP). We name this modified OptBWTR, *OptBWTRL* (i.e. OptBWTR for long LEMs or OptBWTR with LCP). OptBWTRL maintains the O(r) words space complexity of OptBWTR and computes ϕ[i] and PLCP[i] in constant time. The long LEM finding algorithm also requires as input an OptBWTRL of the text. We also discuss possible future work related to this paper, including avenues for improving the results, utilization of constant time PLCP computation to speed up matching statistics computation, and biological applications of long LEMs. Long LEMs may have many biological applications, from identity by descent segment detection and local ancestry inferences, to seeds or anchors for approximate matching algorithms for genome to genome alignment, genome to pangenome, read to genome or other alignments. In this paper, our main contributions are the following:

- **OptBWTRL:** OptBWTRL is an O(r) words space data structure that maintains the capabilities of OptBWTR and adds the ability to compute ϕ, PLCP, and long LEMs efficiently. r is the number of runs in the BWT of the text.
  - **PLCP:** OptBWTRL enables constant time PLCP[i] computation in O(r) space. Note that PLCP[SA[j]]=LCP[j], therefore PLCP computation in constant time allows LCP[j] computation in constant time given SA[j].
  - **Long LEM Query:** We describe an O(m+occ) expected time long LEM query for pattern P and text T given the matching statistics of P with respect to T. The underlying index (OptBWTRL) uses O(r) space. m is the length of P and occ is the number of long LEMs P has with respect to T. A deterministic time bound for a similar algorithm we show is $O\left(m+occ\sqrt{\frac{\text{log}\;occ}{\text{log}\;\text{log}\;occ}}\right)$.
- **Long LEM Query with random access to the text:** Given $O\left(t_{RA}\right)$ time random access to the text and a BWT related index, algorithms for computing matching statistics efficiently are known. Therefore, our long LEM query algorithm results in the following.
  - **In Uncompressed Space:** An algorithm for long LEM query in O(m+occ) expected time in uncompressed string indexes such as the FM Index (Corollary 3.2, variant with O(nσ) space, where n is the length of the text and σ is the size of the alphabet) [18].
  - **In Compressed Space:** An algorithm for long LEM query in $O\left(m\;\text{log}\frac{n}{\delta }+occ\right)$ expected time in $O\left(r+\delta \;\text{log}\frac{n}{\delta }\right)$ space given a block tree [4, 23] (with random access to the text in $O\left(\text{log}\frac{n}{\delta }\right)$ time in $O\left(n\;\text{log}\frac{n}{\delta }\right)$ space) and an OptBWTRL of the text.

## 2 Background

In this section, we review definitions used throughout the rest of the paper. We begin with strings, then in Section 2.1, we review BWT related concepts. In Section 2.2, we give a short overview of the results of Nishimoto and Tabei in [36]. Matching statistics are reviewed in Section 2.3. Finally we review maximal exact matches (MEMs) and define locally maximal exact matches (LEMs) in Section 2.4.

Let Σ={1,2,3,…,σ} be an ordered alphabet of size σ. The size (number of characters it contains) of a string T is represented by |T|. T refers to a text of length n(|T|=n) where the last character is $. The character $ is lexicographically smaller than all other characters in T and occurs only in the last position of T. The i-th character of T is T[i], i∈[1,n]. T[i,j] refers to the substring of T that starts at position i and ends at position j, inclusive (T[i,j]=T[i]T[i+1]T[i+1]…T[j]). Prefix i of T is the string T[1,i], suffix i of T is T[i,n]. The longest common prefix of two strings T and $T^{\prime }$ is referred to by $lcp\left(T,T^{\prime }\right)$. $\left|lcp\left(T,T^{\prime }\right)\right|$ is the largest value i s.t. $i\leq \text{min}\left(|T|,\left|T^{\prime }\right|\right)$ and $T[1,i]=T^{\prime }[1,i]$ (then, $lcp\left(T,T^{\prime }\right)=T[1,i]=T^{\prime }[1,i]$). A string $T^{\prime }$ being lexicographically smaller than T is represented by $T^{\prime }≺T$. If $T^{\prime }=T$, $T^{\prime }⊀T$ and $T⊀T^{\prime }$. If $T^{\prime }\neq T$, $T^{\prime }≺T$ iff $T^{\prime }=lcp\left(T,T^{\prime }\right)$ or $T^{\prime }\left[\left|lcp\left(T,T^{\prime }\right)\right|+1\right]<T\left[\left|lcp\left(T,T^{\prime }\right)\right|+1\right]$.

### 2.1 Burrows-Wheeler Transform

The Suffix Array (SA) of a text T is an array of length n=|T| where the i-th position stores the index of the i-th lexicographically smallest suffix of T. Therefore, T[SA[1],n]≺T[SA[2],n]≺T[SA[3],n]≺⋯≺T[SA[n],n]. The Burrows-Wheeler Transform (BWT) of a text T is a string of length n where the i-th character in the string is the SA[i] – 1-th character of T (the n-th character if SA[i]=1). The LF array is an array of length n that stores the position of the previous suffix in the suffix array, LF[i]=j s.t. SA[j]=SA[i]-1 for all SA[j]∈[1,n-1],LF[i]=j s.t. SA[j]=n for SA[i]=1. The ϕ array stores at position i, the suffix above suffix i in the suffix array, i.e. if SA[k]=i, ϕ[i]=SA[k-1] (ϕ[i]=SA[n] if i=SA[1]). The $\phi^{-1}$ array stores at position i, the suffix below suffix i in the suffix array, i.e. if SA[k]=i, $\phi^{-1}[i]=SA[k+1]$ ($\phi^{-1}[i]=SA[1]$ if i=SA[n]). Therefore, $\phi \left[\phi^{-1}[i]\right]=i$ and $\phi^{-1}[\phi [i]]=i$. The LCP array is an array of length n where LCP[i] stores the length of the longest common prefix of suffix SA[i] and SA[i-1]. LCP[1]=0 and for i∈[2,n], LCP[i]=|lcp(T[SA[i],n],T[SA[i-1],n])|. The PLCP (permuted LCP) array is an array of length n where the LCP array is stored by suffix index. Therefore, if SA[i]=j, PLCP[j]=LCP[i]=|lcp(T[j,n],T[ϕ[j],n])|. Finally, the inverse suffix array, ISA, is an array of length n that stores at position i the position of suffix i in the suffix array, if ISA[i]=j, SA[j]=i. ISA[SA[i]]=i and SA[ISA[i]]=i. The SA, LF, ϕ, $\phi^{-1}$, and ISA arrays are permutations of the integers in [1,n]. SA and ISA are inverses of each other and ϕ and $\phi^{-1}$ are inverses of each other.

**Figure 1:**
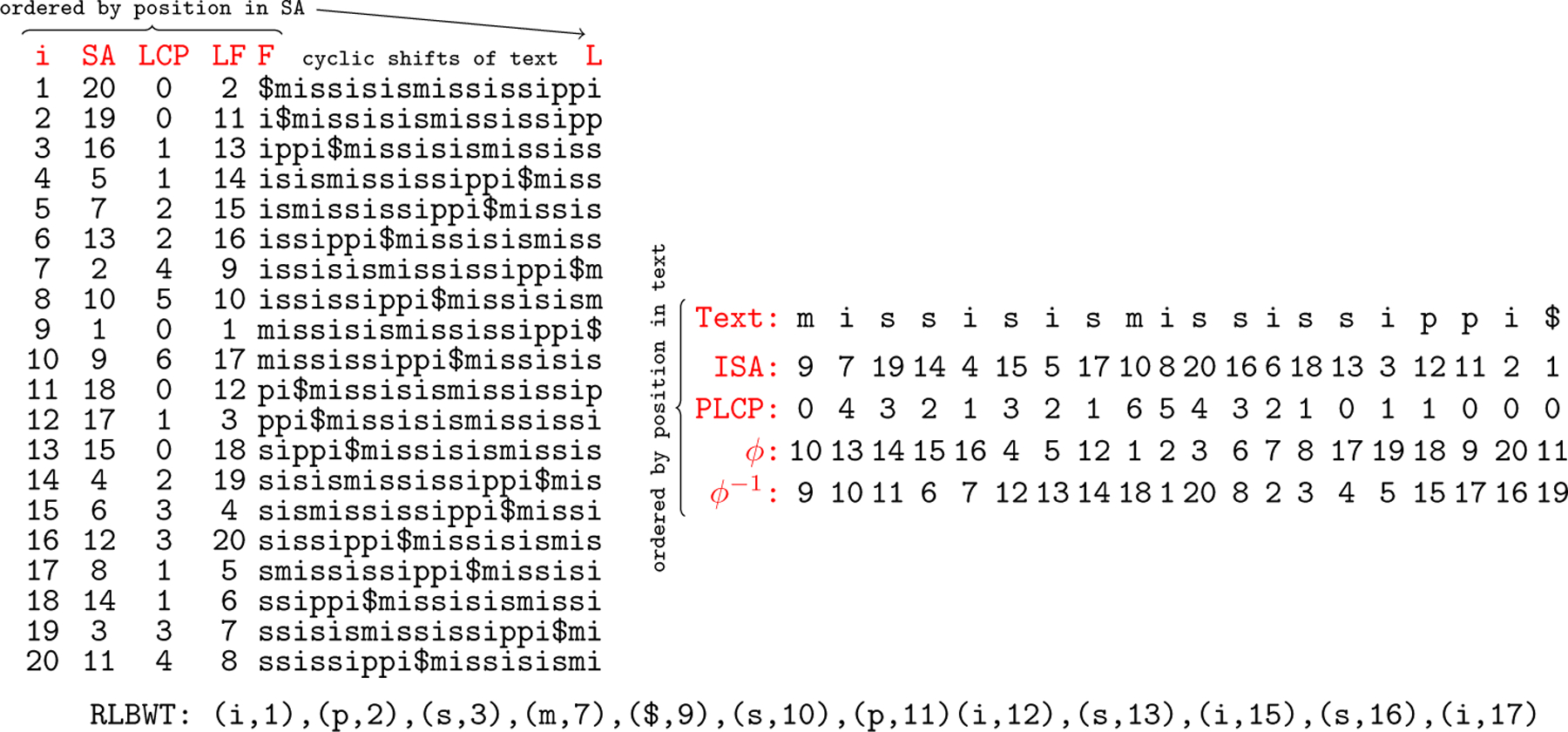
BWT and related structures for T=missisismississippi$. SA,LCP,LF,F,andL are ordered by position in SA while $ISA,\text{PLCP},\phi ,\text{and}\;\phi^{-1}$ are ordered by position in the text.

The run-length Burrows-Wheeler Transform (RLBWT) is the run-length encoding of the BWT of a text. Call L the BWT of text T. Then, L is partitioned into r nonempty substrings $L_{1},L_{2},\ldots ,L_{r}$. $L_{i}$ is a substring of L corresponding to the i-th run of L. A run is a maximal repetition of the same character in L. Therefore, $L_{i}[1]=L_{i}[2]=\cdots =L_{i}\left[\left|L_{i}\right|\right]$ for all i∈[1,r] and $L_{i}[1]\neq L_{i+1}[1]$ for all i∈[1,r-1]. $l_{i}$ is the starting position of the run $L_{i}$ in L. The RLBWT is represented as r pairs $\left(L_{i}[1],l_{i}\right)$ for i∈[1,r]. All of these structures can be seen in Figure 1 for a text T=missisismississippi$.

### 2.2 Move Data Structure

The move data structure is a data structure for representing a permutation of a contiguous range of integers efficiently. It was introduced by Nishimoto and Tabei [36]. In the original introduction, the structure was described for a permutation of [1,n]. This of course may be extended to any bijective function from a contiguous range of integers to another contiguous range of integers. The move data structure takes space proportional to the number of intervals conserved in the function. An interval is conserved in a bijective function from a contiguous range of integers to another contiguous range of integers if for any i, j in the interval, f(i)-f(j)=i-j (therefore, f(i)=f(j)+i-j and f(i)-i=f(j)-j). The move data structure computes the represented function in constant time. The important arrays, LF and $\phi^{-1}$, are permutations of [1,n] with O(r) conserved intervals, where r is the number of runs in the BWT. Therefore, Nishimoto and Tabei define the OptBWTR data structures using move data structures. OptBWTR supports efficient count and locate queries in BWT-runs compressed space. Below, we more formally review some of the results from their paper [36].

#### 2.2.1 Disjoint Interval Sequence

$I=\left(p_{1},q_{1}\right),\left(p_{2},q_{2}\right),\ldots ,\left(p_{k},q_{k}\right)$ is a sequence of k pairs of integers. Let $p_{k+1}=n+1$. Then i is a *disjoint interval sequence* iff there exists a permutation π of [1,k] s.t. (i) $p_{1}=1<p_{2}<\cdots <p_{k}\leq n$, (ii) $q_{\pi [1]}=1$, and (iii) $q_{\pi [i]}=q_{\pi [i-1]}+\left(p_{\pi [i-1]+1}-p_{\pi [i-1]}\right)$. $\left[p_{i},p_{i+1}-1\right]$ is referred to as the i-th input interval, and $\left[q_{i},q_{i}+\left(p_{i+1}-p_{i}\right)-1\right]$ as the i-th output interval. The input intervals don’t overlap, and their union is [1,n]. The output intervals don’t overlap and their union is [1,n].

A *move query* on a disjoint interval sequence I takes as input (i,x), where i is an index in [1,n] and x is the index of the input interval sequence that contains it, i∈[1,n] and $p_{x}\leq i<p_{x+1}$ and x∈[1,k]. The move query outputs $\left(i^{\prime },x^{\prime }\right)$ where $i^{\prime }=q_{x}+\left(i-p_{x}\right)$ and $p_{x^{\prime }}\leq i^{\prime }<p_{x^{\prime }+1}$, i.e. $i^{\prime }$ is the mapping of position i from the input to output intervals by I and $x^{\prime }$ is the index of the input interval that contains $i^{\prime }$. f, a permutation of [1,n] with k conserved intervals, can be represented by a disjoint interval sequence where the input intervals are the conserved intervals and the output intervals are the mapping of the input intervals by f. Then, a move query of (i,x) returning $\left(i^{\prime },x^{\prime }\right)$ computes f by $f(i)=i^{\prime }$.

Nishimoto and Tabei show that move queries on a disjoint interval sequence of k input intervals (and therefore k output intervals) can be computed in constant time and O(k) space with the move data structure. The move data structure is built by splitting the k input intervals of I into at most 2k intervals. This results in a disjoint interval sequence of at most 2k input intervals (and an equivalent number of output intervals) that represents the same permutation as the original disjoint interval sequence. The split interval sequence of i that the move data structure is built on is referred to as a balanced interval sequence. The notation for a balanced interval sequence of I is B(I), and the notation for a move data structure of I is F(I). In this paper, we occasionally use input interval of F(I) as shorthand for input interval of B(I) (for example, i-th input interval of a move data structure refers to the i-th input interval of the balanced interval sequence it was built on). Brown et al. extend the balanced interval sequence result of Nishimoto and Tabei to splitting I’s k intervals into at most $k+\frac{k}{d-1}$ intervals, resulting in a move data structure with O(d) time move query computation for any d≥2 [9].

#### 2.2.2 OptBWTR

The arrays LF and $\phi^{-1}$ are permutations of [1,n] with O(r) conserved intervals. For LF, a conserved interval is within a run in the BWT. For $\phi^{-1}$, a conserved interval is a range of suffixes of T that don’t occur at the bottom of a run in the BWT (except the first position of the interval may be at the bottom of a run). Therefore, Nishimoto and Tabei define the OptBWTR data structure as the combination of the move data structures of the LF and $\phi^{-1}$ functions along with a rank-select data structure on an O(r) length string $L_{\text{first}}$. OptBWTR supports $O\left(m\;\text{log}\;{\text{log}}_{w}\sigma \right)$ time count queries and $O\left(m\;\text{log}\;{\text{log}}_{w}\;\sigma +occ\right)$ time locate queries in O(r) words of space, where r is the number of runs in the BWT of the text, m is the length of the pattern, occ is the number of occurrences of the pattern in the text, w is the word size, σ is the size of the alphabet (Theorem 9 of [36]). The input intervals of $B\left(I_{LF}\right)$, the disjoint interval sequence of the move data structure of LF, are contained within a run in the BWT. Call the i-th input interval of $B\left(I_{LF}\right)\left[p_{i},p_{i+1}-1\right]$. Then, $L_{\text{first}}=L\left[p_{1}\right]L\left[p_{2}\right]L\left[p_{3}\right]\ldots L\left[p_{k}\right]$, where k≤2r is the number of input intervals of $B\left(I_{LF}\right)$, L is the BWT of T, and ∀i∈[1,k], $j\in \left[p_{i},p_{i+1}-1\right]L[j]=L\left[p_{i}\right]$. Call $B\left(I_{SA}\right)$ the disjoint interval sequence of the move data structure of $\phi^{-1}$, and $\left[p_{i}^{-},p_{i+1}^{-}\right]$ its i-th input interval. OptBWTR is composed of:

- move data structures for LF and $\phi^{-1}\left(F\left(I_{LF}\right)\right.$ and $F\left(I_{SA}\right)$ respectively),
- a rank-select data structure on $L_{\text{first}}\left(R\left(L_{\text{first}}\right)\right)$,
- samples of the SA at the beginning of input intervals of the LF move data structure ($SA^{+}$, where $SA^{+}[i]=SA\left[p_{i}\right]$), and
- the index of the input interval of the $\phi^{-1}$ move data structure that contains each SA sample in $SA^{+}$ ($SA_{\text{index}}^{+}$, where $SA_{\text{index}}^{+}=y⟺SA^{+}[i]\in \left[p_{y}^{-},p_{y+1}^{-}\right]$).

### 2.3 Matching Statistics

The matching statistics of a pattern P with respect to a text T represents information on the local similarity of the pattern to the text. The matching statistics of P with respect to T, MPST, is an array of length |P|=m that stores at position i three values: MPSTi.len, MPSTi.suff, and MPSTi.row. MPSTi.len is the length of the longest substring of P starting at i that occurs in T. MPSTi.suff is a suffix of T that has a longest common prefix with P[i,m] of length MPSTi.len (or equivalently, MPSTi.suff is the starting position of an occurrence of Pi,i+MPSTi.len−1 in T). MPSTi.row is the index in the SA of T that has value MPST.suff. Formally for all i∈[1,m],

- MPSTi.len=maxj∈1,TlcpPi,m,Tj,T,
- lcpTMPSTi.suff,n,Pi,m=MPSTi.len, and
- SAMPSTi.row=MPSTi.suff.

When P and T are clear from the context, we omit them from MPST and refer to the matching statistics of P with respect to T as MS.

### 2.4 Maximal and Locally Maximal Exact Matches

For a pattern P and a text T(|P|=m,|T|=n), a maximal exact match (MEM), $P[i,j]=T\left[i^{\prime },j^{\prime }\right]$, is a match between P and T that cannot be extended left or right in the pattern. Formally, (i=1 or P[i-1,j] doesn’t occur in T) and (j=m or P[i,j+1] doesn’t occur in T). A MEM can be fully specified by the triple $\left(i,i^{\prime },k\right)$ where k=j-i+1 is the length of the match and i and $i^{\prime }$ are the starting positions of the match in the pattern and the text respectively.

**Figure 2:**
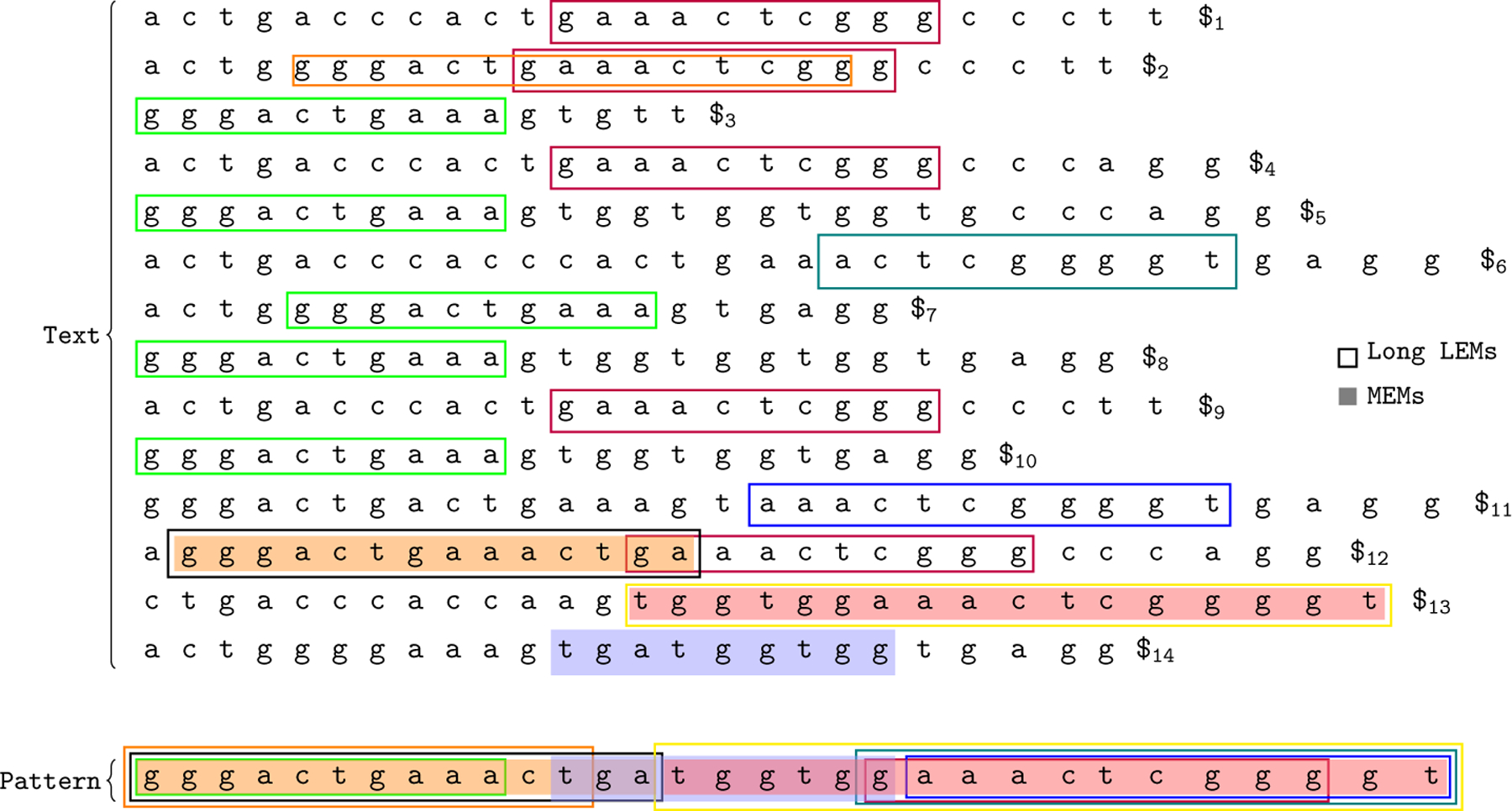
MEMs and LEMs of a pattern (haplotype) vs a text (pangenome). Haplotype i is the sequence of characters between ${\$}_{i-1}$ and ${\$}_{i}$. The text is the concatenation of the haplotypes $T=“\text{actgacccactgaaactcgggccctt}{\$}_{1}\text{actggggactgaaactcgggccctt}{\$}_{2}\ldots ”$. MEMs and long LEMs (length threshold for long LEMs: ℒ=10) of the pattern (a haplotype not contained in the pangenome) with respect to the text (the pangenome) are highlighted. MEMs are shaded in while LEMs are boxed in. In this example, MEMs are only able to detect relationships among the haplotypes most closely related to the pattern haplotype. Haplotypes similar to the pattern but not maximally similar at any location remain undetected. Notably, haplotype 2 is very similar to the pattern but doesn’t contain any MEMs with it. The number of undetected similar haplotypes in biobank scale haplotype panels may be an order of magnitude larger.

For a pattern P and a text T, a locally maximal exact match (LEM), $P[i,j]=T\left[i^{\prime },j^{\prime }\right]$, is a match between P and T that cannot be simultaneously extended in the pattern and the text. The match cannot be simultaneously extended left in the pattern and the text. Likewise, it cannot be simultaneously extended right in the pattern and the text. Formally, (i=1 or $i^{\prime }=1$ or $P[i-1,j]\neq T\left[i^{\prime }-1,j^{\prime }\right]$) and (j=m or $j^{\prime }=n$ or $P[i,j+1]\neq T\left[i^{\prime },j^{\prime }+1\right]$). A LEM can also be fully specified by the triple $\left(i,i^{\prime },k\right)$ where k is the length of the LEM and k=j-i+1. For some length threshold ℒ, a long LEM is a LEM with length at least ℒ. See Figure 2 for a depiction of MEMs and LEMs in a text representing a pangenome.

## 3 Methods

Here we describe the main results of our paper. In Section 3.1, we prove move data structures can compute ϕ and PLCP in constant time. Then we describe OptBWTRL, our modification of OptBWTR that utilizes these move data structures. In Section 3.2, we describe multiple algorithms for long LEM query provided an OptBWTRL of the text and matching statistics of the pattern with respect to the text.

### 3.1 Computing LCP with Move Data Structures

We define $p_{j}^{+}$ to be the j-th smallest suffix that occurs at the top of a run in the BWT. Therefore let (i) $p_{1}^{+}<p_{2}^{+}<\cdots <p_{r}^{+}<p_{r+1}^{+}=n+1$ and (ii) $\left\{p_{1}^{+},p_{2}^{+},\ldots ,p_{r}^{+},p_{r+1}^{+}\right\}=\left\{SA\left[l_{1}\right],SA\left[l_{2}\right],\ldots ,SA\left[l_{r}\right],n+1\right\}$. Lemma 1 and its proof are phrased very similarly to Lemma 4 in [36] to demonstrate its derivativeness and the similarity of the properties.

**Lemma 1.** *(i) Let* x *be the integer satisfying* $p_{x}^{+}\leq i<p_{x+1}^{+}$ *for some integer* i∈[1,n]. *Then* $LCP[ISA[i]]=LCP\left[ISA\left[p_{x}^{+}\right]\right]-\left(i-p_{x}^{+}\right)$.

**Proof.** Lemma 1(i) clearly holds for $i=p_{x}^{+}$. We show that Lemma 4(i) holds for $i\neq p_{x}^{+}$ (i.e., $i>p_{x}^{+}$). Let $s_{t}$ be the position in SA with sa-value $p_{x}^{+}+t$ for an integer t∈[1,y] (i.e., $\left.SA\left[s_{t}\right]=p_{x}^{+}+t\right)$ where $y=i-p_{x}^{+}$. Two adjacent positions $s_{t}-1$ and $s_{t}$ are contained in an interval $\left[l_{v},l_{v}+\left|L_{v}\right|-1\right]$ on LCP which corresponds to the v-th run $L_{v}$ of L. This is because $s_{t}$ is not the starting position of a run, i.e., $\left(SA\left[s_{t}\right]=p_{x}^{+}+t\right)\notin \left\{p_{1}^{+},p_{2}^{+},\ldots ,p_{r}^{+}\right\}$. The LF function maps $s_{t}$ to $s_{t-1}$, where $s_{0}$ is the position with sa-value $p_{x}^{+}$. LF also maps $s_{t}-1$ to $s_{t-1}-1$ by Lemma 3(i) of [36]. $LCP\left[s_{t-1}\right]=LCP\left[s_{t}\right]+1$ due to $s_{t}$ and $s_{t}-1$ being in the same interval on L, $L_{v}$. These relationships produce y equalities $LCP\left[s_{0}\right]=LCP\left[s_{1}\right]+1,LCP\left[s_{1}\right]=LCP\left[s_{2}\right]+1,\ldots ,LCP\left[s_{y-1}\right]=LCP\left[s_{y}\right]+1$. The equalities lead to $LCP\left[s_{0}\right]=LCP\left[s_{y}\right]+y$, and therefore $LCP\left[s_{y}\right]=LCP\left[s_{0}\right]-y$. Which represents $LCP[ISA[i]]=LCP\left[ISA\left[p_{x}^{+}\right]-\left(i-p_{x}^{+}\right)\right.$ by $ISA[i]=s_{y},ISA\left[p_{x}^{+}\right]=s_{0}$, and $y=\left(i-p_{x}^{+}\right)$. ◀

**Lemma 2.** *(i) Let* x *be the integer satisfying* $p_{x}^{+}\leq i<p_{x+1}^{+}$ *for some integer* i∈[1,n]. *Then* $\text{PLCP}[i]=\text{PLCP}\left[p_{x}^{+}\right]-\left(i-p_{x}^{+}\right)$.

**Proof.** By Lemma 1 and PLCP[j]=LCP[ISA[j]] for all j∈[1,n] [21]. ◀

#### 3.1.1 Move Data Structure for ϕ

$p_{j}^{+}$ remains as defined in the previous section. Let $\delta^{+}$ be a permutation of [1,r] satisfying $\phi \left(p_{\delta^{+}[1]}^{+}\right)<\phi \left(p_{\delta^{+}[2]}^{+}\right)<\cdots <\phi \left(p_{\delta^{+}[r]}^{+}\right)$. ϕ has the following properties on RLBWT.

**Lemma 3.** *The following three statements hold: (i) Let* x *be the integer satisfying* $p_{x}^{+}\leq i<p_{x+1}^{+}$ *for some integer* i∈[1,n]. *Then* $\phi (i)=\phi \left(p_{x}^{+}\right)+\left(i-p_{x}^{+}\right)$*; (ii)* $\phi \left(p_{\delta^{+}[1]}^{+}\right)=1$ *and* $\phi \left(p_{\delta^{+}[i]}^{+}\right)=\phi \left(p_{\delta^{+}[i-1]}^{+}\right)+d$ *where* $d=p_{\delta^{+}[i-1]+1}^{+}-p_{\delta^{+}[i-1]}^{+}$*; (iii)* $p_{1}^{+}=1$.

**Proof.** See Appendix A. ◀

We can compute ϕ by using a move data structure. A sequence $I_{\phi }$ consists of r pairs $\left(p_{1}^{+},\phi \left(p_{1}^{+}\right)\right),\left(p_{2}^{+},\phi \left(p_{2}^{+}\right)\right),\ldots ,\left(p_{r}^{+},\phi \left(p_{r}^{+}\right)\right)$. $I_{\phi }$ satisfies the three conditions of a disjoint interval sequence by Lemma 3, and ϕ is equal to the bijective function represented by $I_{\phi }$.

**Lemma 4.** *(i)* $I_{\phi }$ *is a disjoint interval sequence. (ii)* ϕ *is equal to the bijective function represented by* $I_{\phi }$.

**Proof.** (i) $I_{\phi }$ has the following three properties: (a) $p_{1}^{+}=1<p_{2}^{+}<\cdots <p_{r}^{+}\leq n$ holds by Lemma 3(iii) and the definition of the sequence $p_{1}^{+},p_{2}^{+},\ldots ,p_{r+1}^{+}$, (b) $\phi \left(p_{\delta^{+}[1]}^{+}\right)=1$ by Lemma 3(ii), and (c) $\phi \left(p_{\delta^{+}[i]}^{+}\right)=\phi \left(p_{\delta^{+}[i-1]}^{+}\right)+\left(p_{\delta^{+}[i-1]+1}^{+}-p_{\delta^{+}[i-1]}^{+}\right)$. Therefore $I_{\phi }$ satisfies the three conditions of the disjoint interval sequence.

(ii) Let $f_{\phi }$ be the bijective function represented by $I_{\phi }$. Then $f_{\phi }(i)=\phi \left(p_{x}^{+}\right)+\left(i-p_{x}^{+}\right)$ where x is the integer such that $p_{x}^{+}\leq i<p_{x+1}^{+}$ holds. On the other hand, $\phi (i)=\phi \left(p_{x}^{+}\right)+\left(i-p_{x}^{+}\right)$ holds by Lemma 3(i). Therefore $f_{\phi }(i)=\phi (i)$ and $f_{\phi }$ and ϕ are the same function. ◀

Let $F\left(I_{\phi }\right)$ be the move data structure built on the balanced interval sequence $B\left(I_{\phi }\right)$ for $I_{\phi }$. By Lemma 6 of [36], $F\left(I_{\phi }\right)$ requires O(r) words of space. By the results of Section 3.2 of [36], evaluation of a move query using a move data structure for a balanced disjoint interval sequence takes constant time. Finally, $\phi (i)=i^{\prime }$ holds for a move query $\text{Move}\left(B\left(I_{\phi }\right),i,x\right)=(i^{\prime },x^{\prime })$ by Lemma 4. Therefore we have proved (i) of the following lemma.

**Lemma 5.** *(i) There exists a move data structure* $F\left(I_{\phi }\right)$ *that computes* ϕ(i) *in* O(r) *space and constant time given* x, *the index of the input interval of* $I_{\phi }$ *that contains* i. *(ii) This move data structure can be modified to also compute* PLCP[i] *in* O(r) *space and constant time given* i *and* x. *Call the modified move data structure* $F\left(I_{\phi ,\text{PLCP}}\right)$.

**Proof.** Say that $B\left(I_{\phi }\right)$ has $k^{+}$ input intervals and the i-th input interval is $\left[p_{i}^{+},p_{i+1}^{+}\right]$. Then we modify the move data structure $F\left(I_{\phi }\right)$ by adding an array $LCP^{+}$ of size $k^{+}$. $LCP^{+}[i]$ stores the value $LCP\left[ISA\left[p_{x}^{+}\right]\right]$ for each $x\in \left[1,k^{+}\right]$. $\left(\text{PLCP}\left[p_{x}^{+}\right]=LCP\left[ISA\left[p_{x}^{+}\right]\right].\right)\text{PLCP}[i]=\text{PLCP}\left[p_{x}^{+}\right]-\left(i-p_{x}^{+}\right)$ by Lemma 1. Therefore PLCP[i] is computed in constant time by evaluating $LCP^{+}[x]-\left(i-p_{x}^{+}\right)$. Call this modified move data structure $F\left(I_{\phi ,\text{PLCP}}\right)$. ◀

A similar function that we may need to compute is LCP[i+1] given SA[i], i.e. given SA[i]=y, compute $\left|lcp\left(T[y,n],T\left[\phi^{-1}(y),n\right]\right)\right|=\text{PLCP}\left[\phi^{-1}(y)\right]=LCP[i+1]$. Nishimoto and Tabei described $F\left(I_{SA}\right)$, a move data structure computing $\phi^{-1}$. $F\left(I_{SA}\right)$ can be modified to compute $\text{PLCP}\left[\phi^{-1}(y)\right]$ in constant time as well in a similar fashion to the modification of the $F\left(I_{\phi }\right)$ data structure. Call the disjoint interval sequence $F\left(I_{SA}\right)$ is built on $B\left(I_{SA}\right)$. Call the i-th input interval of $B\left(I_{SA}\right)\left[p_{i}^{-},p_{i+1}^{-}-1\right]$, where $B\left(I_{SA}\right)$ has $k^{-}$ input intervals and $p_{k^{-}+1}^{-}=n+1$. Note that by the construction of Nishimoto and Tabei, every suffix at the bottom of a BWT run is the start of an input interval, $\left\{SA\left[l_{2}-1\right],SA\left[l_{3}-1\right],SA\left[l_{4}-\right.\right.\left.1],\ldots ,SA\left[l_{r}-1\right],SA[n]\right\}\subseteq \left\{p_{1}^{-},p_{2}^{-},\ldots ,p_{k}^{-}\right\}$. Then for any $i,j\in \left[p_{x}^{-},p_{x+1}^{-}-1\right],\phi^{-1}(i)-\phi^{-1}(j)=i-j$. Therefore $\phi^{-1}(i)=\phi^{-1}(j)+(i-j)$ (see Lemma 4 in [36]). Below, we prove (ii) that for any $i,j\in \left[p_{x}^{-},p_{x+1}^{-}-1\right],\text{PLCP}\left[\phi^{-1}(i)\right]-\text{PLCP}\left[\phi^{-1}(j)\right]=j-i$, therefore $\text{PLCP}\left[\phi^{-1}(i)\right]=\text{PLCP}\left[\phi^{-1}(j)\right]+j-i$.

**Lemma 6.** *Let* x *be the integer satisfying* $p_{x}^{-}\leq i<p_{x+1}^{-}$ *for some* i∈[1,n]. *Then (i)* $\text{PLCP}\left[\phi^{-1}(i)\right]=\text{PLCP}\left[\phi^{-1}\left(p_{x}^{-}\right)\right]-\left(i-p_{x}^{-}\right)$. *Therefore, (ii) for any* $i,j\in \left[p_{x}^{-},p_{x+1}^{-}-1\right]$, $\text{PLCP}\left[\phi^{-1}(i)\right]=\text{PLCP}\left[\phi^{-1}\left(p_{x}^{-}\right)\right]-\left(i-p_{x}^{-}\right)$, $\text{PLCP}\left[\phi^{-1}(j)\right]=\text{PLCP}\left[\phi^{-1}\left(p_{x}^{-}\right)\right]-\left(j-p_{x}^{-}\right)$, *and* $\text{PLCP}\left[\phi^{-1}(i)\right]-\text{PLCP}\left[\phi^{-1}(j)\right]=j-i$.

**Proof.** Lemma 6(i) clearly holds for $i=p_{x}^{-}$. We show that Lemma 6(i) holds for $p_{x}^{-}<i<p_{x+1}^{-}$ (i.e. $i\neq p_{x}^{-}$). Let $s_{t}$ be the position in the SA with sa-value $p_{x}^{-}+t$ for an integer t∈[1,y] where $y=i-p_{x}^{-}$. Two adjacent positions $s_{t}$ and $s_{t}+1$ are contained in an interval $\left[l_{v},l_{v+1}-1\right]$ corresponding to the v-th run in the BWT $\left(L_{v}\right)$. This is because $s_{t}$ is not the ending position of a run, $SA\left[s_{t}\right]\notin \left\{p_{1}^{-},p_{2}^{-},\ldots ,p_{k^{-}}^{-}\right\}$. The LF function maps $s_{t}$ to $s_{t-1}$, where $s_{0}$ is the position in the SA with value $p_{x}^{-}$. LF also maps $s_{t}+1$ to $s_{t-1}+1$ by Lemma 3(i) of [36]. $\text{PLCP}\left[\phi^{-1}\left(p_{x}^{-}+t-1\right)\right]=LCP\left[s_{t-1}+1\right]=LCP\left[s_{t}+1\right]+1=\text{PLCP}\left[\phi^{-1}\left(p_{x}^{-}+t\right)\right]+1$ since $s_{t}$ and $s_{t}+1$ are in the same interval in the BWT, $L_{v}$. These relationships produce y equalities $\text{PLCP}\left[\phi^{-1}\left(p_{x}^{-}\right)\right]=\text{PLCP}\left[\phi^{-1}\left(p_{x}^{-}+1\right)\right]+1,\text{PLCP}\left[\phi^{-1}\left(p_{x}^{-}+1\right)\right]=\text{PLCP}\left[\phi^{-1}\left(p_{x}^{-}+2\right)\right]+1,\ldots ,\text{PLCP}\left[\phi^{-1}\left(p_{x}^{-}+y-1\right)\right]=\text{PLCP}\left[\phi^{-1}\left(p_{x}^{-}+y\right)\right]+1$. This leads to $\text{PLCP}\left[\phi^{-1}\left(p_{x}^{-}\right)\right]=\text{PLCP}\left[\phi^{-1}\left(p_{x}^{-}+y\right)\right]+y$. Which leads to $\text{PLCP}\left[\phi^{-1}(i)\right]=\text{PLCP}\left[\phi^{-1}\left(p_{x}^{-}\right)\right]-\left(i-p_{x}^{-}\right)$ by $y=i-p_{x}^{-}$ and $p_{x}^{-}+y=i$. ◀

Therefore, the move data structure that computes $\phi^{-1}(i)$, $F\left(I_{SA}\right)$, can be modified to compute $\text{PLCP}\left[\phi^{-1}(i)\right]$ as well.

**Lemma 7.** $F\left(I_{SA}\right)$ *can be modified to compute* $\text{PLCP}\left[\phi^{-1}(i)\right]$ *as well as* $\phi^{-1}(i)$ *in constant time and* O(r) *space given* x, *the index of the input interval of* $B\left(I_{SA}\right)$ *that contains* i. *Call the modified move data structure* $F\left(I_{\phi^{-1},\text{PLCP}}\right)$.

**Proof.** We modify the $F\left(I_{SA}\right)$ move data structure by $LCP^{-}$, an array of size $k^{-}$ where the x-th element stores the value $\text{PLCP}\left[\phi^{-1}\left(p_{x}^{-}\right)\right]$. Then, $\text{PLCP}\left[\phi^{-1}(i)\right]$ can be computed in constant time by evaluating $LCP^{-}[x]-\left(i-p_{x}^{-}\right)$ by $LCP^{-}[x]=\text{PLCP}\left[p_{x}^{-}\right]$ and Lemma 6(i). We call this modified move data structure $F\left(I_{\phi^{-1},\text{PLCP}}\right)$. ◀

#### 3.1.2 OptBWTRL

We slightly modify OptBWTR by adding a move data structure that computes ϕ and PLCP and arrays that allow jumping to the closest input intervals corresponding to adjacent runs in the BWT in constant time. We call it *OptBWTRL*, L for LCP and ℒ long LEMs. In addition to the structures of OptBWTR, OptBWTRL contains $F\left(I_{\phi ,\text{PLCP}}\right)$, ND, PD, $SA^{-}$, $SA_{\phi }^{+}$, $SA_{\text{index}}^{-}$, and $SA_{\phi }^{-}$. Furthermore, the $F\left(I_{SA}\right)$ move data structure of OptBWTR is replaced by the $F\left(I_{\phi^{-1},\text{PLCP}}\right)$ move data structure described in Lemma 7. Recall that $B\left(I_{LF}\right)$ is the disjoint interval sequence the move data structure $F\left(I_{LF}\right)$ is built on. Let $B\left(I_{LF}\right)$ contain k input intervals where the i-th input interval is $\left[p_{i},p_{i+1}-1\right]$, and $p_{k+1}=n+1$. Further recall that every input interval is contained in a run in the BWT, i.e. for all i∈[1,k], $\forall j,j^{\prime }\in \left[p_{i},p_{i+1}-1\right]$, $L[j]=L\left[j^{\prime }\right]$. Then, ND and PD are arrays of length k where ND contains the index of the next input interval with a different character in the BWT and PD contains the index of the previous input interval with a different character in the BWT. Formally, for all i∈[1,k], $ND[i]=\text{min}\left\{j>i|L\left[p_{i}\right]\neq L\left[p_{j}\right]\right\}$, and $PD[i]=\text{max}\left\{j<i|L\left[p_{i}\right]\neq L\left[p_{j}\right]\right\}$. If no such j exists, ND[i]=k+1 and PD[i]=-1. ND and PD can be constructed in O(k) (and therefore, O(r)) time given $L_{\text{first}}$. $SA^{-}$ are samples of the SA at the ends of input intervals of $B\left(I_{LF}\right)$. $SA_{\phi }^{+}$ are the indices of the input intervals of the top of $B\left(I_{LF}\right)$ input interval suffix array samples in $F\left(I_{\phi ,\text{PLCP}}\right)$. $SA_{\text{index}}^{-}$ and $SA_{\phi }^{-}$ are the indices of the input intervals of the bottom of $B\left(I_{LF}\right)$ input interval suffix array samples in $F\left(I_{\phi^{-1},\text{PLCP}}\right)$ and $F\left(I_{\phi ,\text{PLCP}}\right)$ respectively. Below, let $\left[p_{i}^{+},p_{i+1}^{+}-1\right]$ and $\left[p_{i}^{-},p_{i+1}^{-}-1\right]$ be the i-th input intervals of $F\left(I_{\phi ,\text{PLCP}}\right)$ and $F\left(I_{\phi^{-1},\text{PLCP}}\right)$ respectively. Then, OptBWTRL differs from OptBWTR in the following ways.

- Replaced $F\left(I_{SA}\right)$ with $F\left(I_{\phi^{-1},\text{PLCP}}\right)$ from Lemma 7.
- Added $F\left(I_{\phi ,\text{PLCP}}\right)$ from Lemma 5.
- Added ND and PD. $ND[i]={\text{min}}_{j>i}\left\{L_{\text{first}}[j]\neq L_{\text{first}}[i]\right.$ or $\left.j=n+1\right\}.PD[i]={\text{max}}_{j>i}\left\{L_{\text{first}}[j]\neq L_{\text{first}}[i]\right.$ or $\left.j=-1\right\}$.
- Added $SA^{-}$. $SA^{-}[i]=SA\left[l_{i+1}-1\right]$.
- Added $SA_{\phi }^{+}$. $SA_{\phi }^{+}[i]=j$ s.t. $p_{j}^{+}\leq SA^{+}[i]<p_{j+1}^{+}$.
- Added $SA_{\text{index}}^{-}.SA_{\text{index}}^{-}[i]=j$ s.t. $p_{j}^{-}\leq SA^{-}[i]<p_{j+1}^{-}$.
- Added $SA_{\phi }^{-}$. $SA_{\phi }^{-}[i]=j$ s.t. $p_{j}^{+}\leq SA^{-}[i]<p_{j+1}^{+}$.

### 3.2 Computing Long LEMs

Here, we describe an algorithm for outputting all the long LEMs of a pattern P with respect to a text T in O(m+occ) expected time using an index of size O(r) words given the matching statistics of P with respect to T and an OptBWTRL of T.m is the length of P and occ is the number of long LEMs P has with T. Furthermore, the matching statistics are slightly augmented to contain the input intervals it’s corresponding data are contained in. In particular, the input interval of $F\left(I_{LF}\right)$ that *MS.row* is contained in is stored as *MS.i*, the input interval of $F\left(I_{\phi ,\text{PLCP}}\right)$ that *MS.suff* is contained in is stored as *MS.w*, and the input interval of $F\left(I_{\phi^{-1},\text{PLCP}}\right)$ that *MS.suff* is contained in is stored as *MS.x*. Note that the long LEM query algorithm we present here does not necessarily result in an O(m+occ) expected time algorithm for outputting all long LEMs of P with respect to T given a OptBWTRL of T because an algorithm for computing the matching statistics of P with respect to T in O(m) time and O(r) space is not known.

We define the *balanced sa*_*lcp*_*-interval* of a string P as a 13-tuple (b,d,e,SA[b],SA[d],SA[e],i,j,k,v,w,x,y) where [b,e] is the sa-interval of P,d∈[b,e],i,j, and k are the indexes of the input intervals of $B\left(I_{LF}\right)$ that contain b,d, and e respectively, v and w are the indexes of the input intervals of $B\left(I_{\phi ,\text{PLCP}}\right)$ containing SA[b] and SA[d] respectively, and x and y are the indexes of the input intervals of $B\left(I_{\phi^{-1},\text{PLCP}}\right)$ of SA[d] and SA[e] respectively. The balanced sa_lcp_-interval keeps track of three positions in the sa-interval: the top (b), bottom (e), and the middle (d). d is any position in the interval, it may be equivalent to the top or the bottom. Each position also maintains its corresponding suffix array value and index of the input interval of the position in $F\left(I_{LF}\right)$ (i,j,and k for top, middle, and bottom respectively). Finally, the top maintains the index of the input interval of its sa-value in $F\left(I_{\phi ,\text{PLCP}}\right)(v)$, the bottom maintains the index of the input interval of its sa-value in $F\left(I_{\phi^{-1},\text{PLCP}}\right)(y)$, and the middle maintains the index of the input interval of its sa-value in both $F\left(I_{\phi ,\text{PLCP}}\right)$ and $F\left(I_{\phi^{-1},\text{PLCP}}\right)$ (w and x respectively). The balanced sa_lcp_-interval of a string P with no occurrences in T is undefined.

The high level idea of the long LEM finding algorithm is to compute the balanced sa_lcp_-interval of adjacent substrings of length ℒ of the pattern while outputting long LEMs along the way. I.E. given the balanced sa_lcp_-interval of P[f+1,f+ℒ], compute the sa_lcp_-interval of P[f,f+ℒ-1] and output all long LEMs of the form $P[f+1,g]=T\left[f^{\prime },g^{\prime }\right]$. We call this problem *long sa*_*lcp*_*-interval advancement*. Given an algorithm for long sa_lcp_-interval advancement in $O\left(t_{ℒ}\right)$ time, a straightforward long LEM computation algorithm is iterating from f=m→1, repeatedly advancing the sa_lcp_-interval and outputting all long LEMs in $O\left(mt_{ℒ}\right)$ time. In Section 3.2.1, we outline an algorithm for balanced sa_lcp_-interval extension and in Section 3.2.2, we outline an algorithm for long sa_lcp_-interval advancement. These algorithms result in an O(m+occ) expected time algorithm for long LEM computation.

#### 3.2.1 Balanced sa_lcp_-interval Extension

Here, we provide algorithms for obtaining the balanced sa_lcp_-interval of cP given the balanced sa_lcp_-interval of P and an OptBWTRL of T. The first algorithm runs in $O\left(\text{log}\;{\text{log}}_{w}\;\sigma \right)$ time by making use of the rank-select structure on $L_{\text{first}}$. The second runs in time linear to the number of runs in the balanced sa_lcp_-interval of $P,r_{P}$, by iterating through them. Call the balanced sa_lcp_-interval of P(b,d,e,SA[b],SA[d],SA[e],i,j,k,v,w,x,y) and the balanced sa_lcp_-interval of $cP\left(b^{\prime },d^{\prime },e^{\prime },SA\left[b^{\prime }\right],SA\left[d^{\prime }\right],SA\left[e^{\prime }\right],i^{\prime },j^{\prime },k^{\prime },v^{\prime },w^{\prime },x^{\prime },y^{\prime }\right)$. Recall that $p_{j},p_{j}^{+},\text{and}\;p_{j}^{-}$ are the starting indexes of the j-th input intervals of $F\left(I_{LF}\right),F\left(I_{\phi ,\text{PLCP}}\right),\text{and}\;F\left(I_{\phi^{-1},\text{PLCP}}\right)$ respectively.

We first discuss the computation of the top values, $b^{\prime },SA\left[b^{\prime }\right],i^{\prime },\text{and}\;v^{\prime }$. If L[b]=c, then $b^{\prime }=LF\left[b\right]\;\text{and}\;i^{\prime }$ can be computed with $F\left(I_{LF}\right)$ in constant time using $\left(b,i\right).SA\left[b^{\prime }\right]=SA\left[b\right]-1,\text{and}\;v^{\prime }=v$ if $SA[b]\neq p_{v}^{+}$, otherwise $v^{\prime }=v-1$. If $L[b]\neq c,b^{\prime }=LF[\overset{ˆ}{b}]$, where $\overset{ˆ}{b}$ is the first location in [b,e] such that $L[\overset{ˆ}{b}]=c$. If $\overset{ˆ}{i}$ is the index of first input interval $i\leq \overset{ˆ}{i}\leq k$ such that $L_{\text{first}}[\overset{ˆ}{i}]=c$, then $\overset{ˆ}{b}=p_{\overset{ˆ}{i}}$, where $p_{a}$ is the starting position of the a-th input interval of $F\left(I_{LF}\right).\overset{ˆ}{i}$ can be computed in $O\left(\text{log}\;{\text{log}\;}_{w}\;\sigma \right)$ time using $R\left(L_{\text{first}}\right)$ or $O\left(r_{P}\right)$ time by iterating through the runs of balanced sa_lcp_-interval of P using the ND array. Then, $i^{\prime }\text{and}\;b^{\prime }=LF[\overset{ˆ}{b}]$ can be computed with $F\left(I_{LF}\right)$ in constant time using $\left(\overset{ˆ}{b},\overset{ˆ}{i}\right)$. $SA\left[b^{\prime }\right]=SA^{+}\left[\overset{ˆ}{i}\right]-1,\text{and}\;v^{\prime }=SA_{\phi }^{+}[\overset{ˆ}{i}]-1$.

The bottom values $e^{\prime },SA\left[e^{\prime }\right],k^{\prime },\text{and}\;y^{\prime }$ can be computed in a similar fashion. If L[e]=c, then $e^{\prime }=LF\left[e\right]\;\text{and}\;k^{\prime }$ can be computed with $F\left(I_{LF}\right)$ in constant time using (e,k). $SA\left[e^{\prime }\right]=SA\left[e\right]-1,\text{and}\;y^{\prime }=y$ if $SA[e]\neq p_{k}^{-}$, otherwise $y^{\prime }=y-1$. If L[e]≠c, then $e^{\prime }=LF[\overset{ˆ}{e}]$, where $\overset{ˆ}{e}$ is the last location in [b,e] such that $L[\overset{ˆ}{e}]=c$. If $\overset{ˆ}{k}$ is the index of the last input input interval $i\leq \overset{ˆ}{k}\leq k$ such that $L_{\text{first}}[\overset{ˆ}{k}]=c$, then $\overset{ˆ}{e}=p_{\overset{ˆ}{k}+1}-1$. $\overset{ˆ}{k}$ can be computed in $O\left(\text{log}\;{\text{log}}_{w}\;\sigma \right)$ time using $R\left(L_{\text{first}}\right)$ or $O\left(r_{p}\right)$ time by iterating through the runs of the balanced sa_lcp_-interval of P using the PD array. Then, $k^{\prime }$ and $e^{\prime }=LF[\overset{ˆ}{e}]$ can be computed with $F\left(I_{LF}\right)$ in constant time using ($\overset{ˆ}{e},\overset{ˆ}{k}$). Finally, $SA\left[e^{\prime }\right]=SA^{-}\left[\overset{ˆ}{k}\right]-1,\text{and}{\;y}^{\prime }=SA_{\text{index}}^{+}[\overset{ˆ}{k}]-1$.

Lastly, the middle values $d^{\prime },SA\left[d^{\prime }\right],j^{\prime },w^{\prime }$ and $x^{\prime }$ need to be computed. Pseudocode for middle value computation is provided as Algorithm 4 in Appendix B. If L[d]=c, then $d^{\prime }=LF[d]$ and $j^{\prime }$ can be computed in constant time with $F\left(I_{LF}\right)$ using (d,j). $SA\left[d^{\prime }\right]=SA[d-1]$. $w^{\prime }=w$ if $SA[d]\neq p_{w}^{+}$, otherwise $w^{\prime }=w-1$. Finally, $x^{\prime }=x$ if $SA[d]\neq p_{x}^{-}$, otherwise $x^{\prime }=x-1$. If L[d]≠c and cP occurs in T, then there is a preceding or succeeding input interval of $B\left(I_{LF}\right)$ that intersects with the balanced sa_lcp_-interval of P and has value c in the BWT. Suppose there is a preceding interval, $\overset{ˆ}{j}$. Then the middle values can be updated similar to the bottom values. Let $\overset{ˆ}{d}=p_{\overset{ˆ}{j}+1}-1$, then $j^{\prime }$ and $d^{\prime }=LF[\overset{ˆ}{d}]$ are computed in constant time with $F\left(I_{LF}\right),SA\left[d^{\prime }\right]=SA^{-}\left[\overset{ˆ}{j}\right]-1,x^{\prime }=SA_{\text{index}}^{-}\left[\overset{ˆ}{j}\right]-1,\text{and}\;w^{\prime }=SA_{\phi }^{-}[\overset{ˆ}{j}]$ if $SA[\overset{ˆ}{d}]\neq p_{SA_{\phi }^{-}[\overset{ˆ}{j}]}^{+}$, otherwise $w^{\prime }=SA_{\phi }^{-}[\overset{ˆ}{j}]-1$. If there is no preceding interval, then set $\overset{ˆ}{j}$ to the index of the succeeding interval. Then the middle values can be updated similar to the top values. Let $\overset{ˆ}{d}=p_{\overset{ˆ}{j}}$, then $j^{\prime }$ and $d^{\prime }=LF[\overset{ˆ}{d}]$ are computed in constant time with $F\left(I_{LF}\right)$, $SA\left[d^{\prime }\right]=SA^{+}\left[\overset{ˆ}{j}\right],w^{\prime }=SA_{\phi }^{+}\left[\overset{ˆ}{j}\right]-1,\text{and}\;x^{\prime }=SA_{\text{index}}^{+}[\overset{ˆ}{j}]$ if $SA[\overset{ˆ}{d}]\neq p_{SA_{\text{index}}^{+}[\overset{ˆ}{j}]}^{-}$, otherwise $x^{\prime }=SA_{\text{index}}^{+}[\overset{ˆ}{j}]-1$. The index, $\overset{ˆ}{j}$, of the preceding or succeeding interval in the sa_lcp_-interval of P with value c in the BWT can be found in $O\left(\text{log}\;{\text{log}}_{w}\;\sigma \right)$ time with $R\left(L_{\text{first}}\right)$ or $O\left(r_{P}\right)$ time by iterating through the runs in the BWT with PD and ND. Therefore, the balanced sa_lcp_-interval of cP can be computed in $O\left(\text{log}\;{\text{log}}_{w}\;\sigma \right)$ time or $O\left(r_{P}\right)$ time given the balanced sa_lcp_-interval of P. See Algorithm 3 in Appendix B for the $O\left(r_{P}\right)$ time algorithm pseudocode.

#### 3.2.2 Long sa_lcp_-interval Advancement

Let $occ_{\text{start},f+1}$ be the number of long LEMs of the form $P\left[f+1,g\right]=T\left[f^{\prime },g^{\prime }\right]\;\text{and}\;occ_{\text{end},f+ℒ-1}$. be the number of long LEMs of the form $P[h,f+ℒ-1]=T\left[f^{\prime \prime },g^{\prime \prime }\right]$. Here, we describe an algorithm that computes the balanced sa_lcp_-interval of P[f,f+ℒ-1] and a dynamic dictionary of the suffixes of T present in the balanced sa_lcp_-interval of P[f,f+ℒ-1]. This algorithm also outputs all $occ_{\text{start},f+1}$ long LEMs of the form $P[f+1,g]=T\left[f^{\prime },g^{\prime }\right]$. The algorithm runs in $O\left(occ_{\text{start},f+1}+occ_{\text{end},f+ℒ-1}\right)$ expected time and requires as input the balanced sa_lcp_-interval of P[f+1,f+ℒ], an OptBWTRL of T, and a dynamic dictionary of the suffixes of T present in the balanced sa_lcp_-interval of P[f+1,f+ℒ].

We begin with the description of the dynamic dictionary, ${\text{dict}}_{occ}$. There are numerous dynamic dictionary data structures that support expected constant time insertion, deletion, and queries [5, 6, 14, 37]. Therefore, we maintain a dynamic dictionary of the suffixes in the balanced sa_lcp_-interval. More precisely, if the balanced sa_lcp_-interval of P[f+1,f+ℒ] is (b,d,e,SA[b],SA[d],SA[e],i,j,k,v,w,x,y), then the dynamic dictionary provided as input to the long sa_lcp_-interval advancement algorithm has e-b+1 elements. ∀a∈[b,e],SA[a]-(f+1) is contained in the dictionary and has the value (f+1)+|lcp(T[SA[a],n],P[f+1,m])|-1=f+|lcp(T[SA[a],n],P[f+1,m])| associated with it. I.E. the value associated with each suffix SA[a] of the text contained in the dictionary is the ending position (in the pattern) of the longest match between suffix SA[a] of the text and suffix f+1 of the pattern. It is not possible that multiple suffixes of T share the same key in ${\text{dict}}_{occ}$. Each suffix of T can occur only once in the dictionary because each suffix of T can occur only once in any balanced sa_lcp_-interval. Each suffix of T can occur only once in any balanced sa_lcp_-interval since each suffix of T occurs exactly once in SA.

Here, we describe the procedure for outputting all $occ_{\text{star}t,f+1}$ long LEMs of the form $P[f+1,g]=T\left[f^{\prime },g^{\prime }\right]$ in $O\left(occ_{\text{star}t,f+1}\right.)$ expected time (we call this *outputMatches*). The high level idea is to iterate through the input intervals of $B\left(I_{LF}\right)$, skipping intervals corresponding to a run of P[f] in constant time per run using ND. We outline two functions: outputMatchesDown(s,ι,z) and outputMatchesUp(s,ι,z). For both functions, s represents a suffix of T and ι is the index of the input interval that contains it in $F\left(I_{\phi^{-1},\text{PLCP}}\right)$ and $F\left(I_{\phi ,\text{PLCP}}\right)$ in *outputMatchesDown* and *outputMatchesUp* respectively. z represents the number of matches to output (directly above s in SA for *outputMatchesUp* and directly below s in SA for *outputMatchesDown*) including s. outputMatchesUp(s,ι,1) outputs a match P[f+1,g]=T[s,s+g-(f+1)], where $g={\text{dict}}_{\text{occ}}[s-(f+1)]$, and removes the key-value pair (s-(f+1),g) from ${\text{dict}}_{\text{occ}}$. outputMatchesUp(s,ι,z) for z>1 similarly outputs a match P[f+1,g]=T[s,s+g-(f+1)] where $g={\text{dict}}_{\text{occ}}[s-(f+1)]$, then removes the key-value pair (s-(f+1),g) from ${\text{dict}}_{\text{occ}}$. Then, it recurses on $\text{outputMatchesUp}\left(s^{\prime },\iota^{\prime },z-1\right)$, where $\iota^{\prime }$ and $s^{\prime }=\phi (s)$ are computed in constant time using $F\left(I_{\phi ,\text{PLCP}}\right)$. outputMatchesDown(s,ι,z) operates in the same way as *outputMatchesDown* except it computes $\phi^{-1}$ instead of ϕ (using $F\left(I_{\phi^{-1},\text{PLCP}}\right)$ instead of $\left.F\left(I_{\phi ,\text{PLCP}}\right)\right)$. It is simple to see that outputMatchesUp(s,ι,z) and outputMatchesDown(s,ι,z) operate in O(z) expected time and output z matches each. Now we utilize *outputMatchesUp* and *outputMatchesDown* to output the $occ_{\text{start},f+1}$ long LEMs of the form $P[f+1,g]=T\left[f^{\prime },g^{\prime }\right]$. If the sa_lcp_-interval of P[f+1,f+ℒ] is fully contained in one input interval of $F\left(I_{LF}\right)$, then i=k. If $L_{\text{first}}[i]=P[f]$, then there are no matches to output, otherwise, every suffix in the balanced sa_lcp_-interval needs to be outputted and we do so by calling outputMatchesUp(SA[d],w,d-b+1) and $\text{outputMatchesDown}\left(\phi^{-1}(SA[d]),x^{\prime },e-b\right)$, where $x^{\prime }$ and $\phi^{-1}(SA[d])$ are computed with (SA[d],x) and $F\left(I_{\phi^{-1},\text{PLCP}}\right)$. In the case where the balanced sa_lcp_-interval of P[f+1,f+ℒ] is not fully contained in one input interval (i≠k), we do the following. For the first input interval, i, if $L_{\text{first}}[i]\neq P[f]$, then the $p_{i+1}-b$ long LEMs starting at $SA\left[p_{i+1}-1\right],SA\left[p_{i+1}-2\right],\ldots ,SA[b]$ in the text are outputted in $O\left(p_{i+1}-b\right)$ expected time by calling $\text{outputMatchesUp}(SA^{-}[i],SA_{\phi }^{-}[i],p_{i+1}-b)$. For any middle input interval o,i<o<j, if $L_{\text{first}}[o]=P[f]$, then this run in the BWT is skipped, o=ND[o]. Otherwise, if $L_{\text{first}}[o]\neq P[f]$, then the long LEMs starting at $SA\left[p_{o}\right],SA\left[p_{o}+1\right],\ldots ,SA\left[p_{o+1}-1\right]$ are outputted by calling $\text{outputMatchesDown}\left(SA^{+}[o],SA_{\text{index}}^{+}[o],p_{o+1}-p_{o}\right)$. For the last input interval, k, if $L_{\text{first}}[k]\neq P[f]$, then the $e-p_{k}+1$ long LEMs starting at $SA\left[p_{k}\right],SA\left[p_{k}+\right.1],\ldots ,SA[e]$ are outputting by calling $\text{outputMatchesDown}\left(SA^{+}[k],SA_{\text{index}}^{+}[k],e-p_{k}+1\right)$. Overall, outputting the $occ_{\text{start},f+1}$ long LEMs of the form $P[f+1,g]=T\left[f^{\prime },g^{\prime }\right]$ takes $occ_{\text{start},f+1}\left.+r_{P[f+1,f+ℒ]}\right)$ expected time. Furthermore, for every run of character P[f] intersecting the sa_lcp_-interval of P[f+1,f+ℒ] except the first one, there is a run of characters ≠ P[f]. Therefore $r_{P[f+1,f+ℒ]}=O\left(occ_{\text{start},f+1}\right)$ Therefore outputting the $occ_{\text{start},f+1}$ long LEMs of the form $P[f+1,g]=T\left[f^{\prime },g^{\prime }\right]$ takes $O\left(occ_{\text{start},f+1}\right)$ expected time. See Algorithms 5–7 in Appendix B for *outputMatches* and related pseudocodes.

Finally, we must compute the balanced sa_lcp_-interval of P[f,f+ℒ-1]. First suppose that the balanced sa_lcp_-interval of P[f+1,f+ℒ] is nonempty. Then, we use the algorithm described in Section 3.2.1 to obtain the sa_lcp_-interval of P[f,f+ℒ] in $O\left(r_{P[f+1,f+ℒ]}\right)$ time. Now, let the sa_lcp_-interval of P[f,f+ℒ] be ($\overset{ˆ}{b},\overset{ˆ}{d},\overset{ˆ}{e},SA[\overset{ˆ}{b}],SA[\overset{ˆ}{d}],SA[\overset{ˆ}{e}],\overset{ˆ}{i},\overset{ˆ}{j},\overset{ˆ}{k},\overset{ˆ}{v},\overset{ˆ}{w},\overset{ˆ}{x},\overset{ˆ}{y}$) and the sa_lcp_-interval of P[f,f+ℒ-1] be ($b^{\prime },d^{\prime },e^{\prime },SA\left[b^{\prime }\right],SA\left[d^{\prime }\right],SA\left[e^{\prime }\right],i^{\prime },j^{\prime },k^{\prime },v^{\prime },w^{\prime },x^{\prime },y^{\prime }$). These sa_lcp_-intervals differ only by those suffixes of the text whose lcp with P[f,m] has length exactly ℒ. There are exactly $occ_{\text{end},f+ℒ-1}$ such suffixes. Furthermore, $\text{PLCP}\left[SA\left[b^{\prime }\right]\right]<ℒ$ and $\text{PLCP}\left[\phi^{-1}\left(SA\left[e^{\prime }\right]\right)\right]<ℒ$. Finally, $\forall b^{\prime }<a\leq \overset{ˆ}{b},LCP[a]=\text{PLCP}[SA[a]]\geq ℒ$, and $\forall \overset{ˆ}{e}\leq a<e^{\prime },LCP[a+1]=\text{PLCP}[SA[a+1]]=\text{PLCP}\left[\phi^{-1}(a)\right]\geq ℒ$. Therefore, we initialize $b^{\prime }=\overset{ˆ}{b},SA\left[b^{\prime }\right]=SA[\overset{ˆ}{b}],i^{\prime }=\overset{ˆ}{i}$, and $v^{\prime }=\overset{ˆ}{v}$. Then, while $LCP\left[b^{\prime }\right]=\text{PLCP}\left[SA\left[b^{\prime }\right]\right]\geq ℒ$, we (i) set $i^{\prime }=i^{\prime }-1$ if $b^{\prime }=p_{i^{\prime }}$, (ii) set $b^{\prime }=b^{\prime }-1$, (iii) update $SA\left[b^{\prime }\right]$ and $v^{\prime }$ by $F\left(I_{\phi ,\text{PLCP}}\right)$, and (iv) insert the key $SA\left[b^{\prime }\right]-f$ into ${\text{dict}}_{occ}$ with value f+ℒ-1. When $LCP\left[b^{\prime }\right]=\text{PLCP}\left[SA\left[b^{\prime }\right]\right]<ℒ$, the final value $b^{\prime }$ has been computed. Similarly for $e^{\prime }$, we initialize $e^{\prime }=\overset{ˆ}{e},SA\left[e^{\prime }\right]=SA[\overset{ˆ}{e}],k^{\prime }=\overset{ˆ}{k}$, and $y^{\prime }=\overset{ˆ}{y}$. Then, while $LCP\left[e^{\prime }+1\right]=\text{PLCP}\left[SA\left[e^{\prime }+1\right]\right]=\text{PLCP}\left[\phi \left(SA\left[e^{\prime }\right]\right)\right]\geq ℒ$, we (i) set $k^{\prime }=k^{\prime }-1$ if $e^{\prime }=p_{k^{\prime }+1}-1$, (ii) set $e^{\prime }=e^{\prime }-1$, (iii) update $SA\left[e^{\prime }\right]$ and $y^{\prime }$ by $F\left(I_{\phi^{-1},\text{PLCP}}\right)$, and (iv) insert the key $SA\left[e^{\prime }\right]-f$ into ${\text{dict}}_{occ}$ with value f+ℒ-1. When $LCP\left[e^{\prime }+1\right]=\text{PLCP}\left[SA\left[e^{\prime }+1\right]\right]=\text{PLCP}\left[\phi^{-1}\left(e^{\prime }\right)\right]<ℒ$, the final value $e^{\prime }$ has been computed. This takes constant time per suffix added to the interval, therefore $O\left(occ_{end,f+ℒ-1}\right)$ time.

If the balanced sa_lcp_-interval of P[f+1,f+ℒ] is empty, the balanced sa_lcp_-interval of P[f,f+ℒ-1] is only nonempty if MS[f].len=ℒ. If it is, we initialize the balanced sa_lcp_-interval of P[f,f+ℒ-1] to $\left(\overset{ˆ}{b}=MS[f].row,\overset{ˆ}{d}=MS[f].row,\overset{ˆ}{e}=MS[f].row,SA[\overset{ˆ}{b}]=MS[f].\text{suff},SA[\overset{ˆ}{d}]=MS[f].\text{suff},SA[\overset{ˆ}{e}]=MS[f].\text{suff},\overset{ˆ}{i}=MS[f].i,\overset{ˆ}{j}=MS[f].i,\overset{ˆ}{k}=MS[f].i,\overset{ˆ}{v}=MS[f].w,\overset{ˆ}{w}=MS[f].w,\overset{ˆ}{x}=MS[f].x,\overset{ˆ}{y}=MS[f].x\right)$ and insert the key MS.suff-f into ${\text{dict}}_{occ}$ with value f+ℒ-1. Then, the interval is expanded to the sa_lcp_-interval of P[f,f+ℒ-1] in $O\left({occ}_{end,f+ℒ-1})\right.$ time as in the other case.

In the case where the balanced sa_lcp_-interval of P[f+1,f+ℒ] is empty, long sa_lcp_-interval advancement is performed in $O\left(occ_{\text{end},f+ℒ-1}\right)$ expected time. If it is not empty, the algorithm we have described first performs sa_lcp_-interval extension, obtaining the sa_lcp_-interval of P[f,f+ℒ] in $O\left(r_{P[f+1,f+ℒ]}\right)$ time and then takes $O\left(occ_{\text{end},f+ℒ-1}\right)$ expected time to compute the sa_lcp_-interval of P[f,f+ℒ-1] from the sa_lcp_-interval of P[f,f+ℒ]. Finally, $r_{P[f+1,f+ℒ]}=O\left(occ_{\text{end},f+ℒ-1}\right)$. Therefore, the algorithm described here performs the long sa_lcp_-interval advancement in $O\left(occ_{\text{start},f+1}+occ_{\text{end},f+ℒ-1}\right)$ expected time. See Algorithm 2 in the Appendix for the pseudocode of this algorithm.

### 3.3 Time Complexity

If the above algorithm is iterated from f=m→0, all long MEMs of the pattern with respect to the text are outputted. The time complexity of the algorithm is the sum of the time complexity of the m long sa_lcp_-interval advancements. Note that the sum of $occ_{\text{start},f+1}$ for f=m→0 is occ and the sum of the $occ_{\text{end},f+ℒ-1}$ for f=m→0 is also occ. Therefore, the time complexity of the algorithm overall is O(m+occ) expected time. See Algorithm 1 in Appendix B for pseudocode. The algorithm takes O(r) space for the OptBWTRL and O(occ) space for maintaining the dynamic dictionary [5]. Also note that if a deterministic time bound is desired, this algorithm runs in $O\left(m+occ\sqrt{\frac{\text{log}\;occ}{\text{log}\;\text{log}\;occ}}\right)$ time with the same space by replacing the dictionary with a deterministic dictionary implemented by exponential search trees [2,47]. Recall these complexities are when given the modified matching statistics. A linear time algorithm for computing matching statistics in O(r) space is not known. However, note that since the values of matching statistics are only needed for positions i where MS[i].len=ℒ, a straightforward algorithm for long LEM query follows from our algorithm in $O\left(mℒ\text{log}\;{\text{log}}_{w}\;\sigma +occ\right)$ expected time when matching statistics are not given as input. This algorithm is obtained by computing the sa_lcp_-interval of each P[i,i+ℒ-1] independently in $O\left(ℒ\text{log}\;{\text{log}}_{w}\;\sigma \right)$ time using the standard count algorithm described by Nishimoto and Tabei [36] followed by performing the long LEM query described here. The long LEM query algorithm described here results in an O(m+occ) expected time long LEM query algorithm in uncompressed string indexes since algorithms for O(m) time matching statistics computation are known in uncompressed space.

## 4 Discussion

In this paper, we have described OptBWTRL, a modification of OptBWTR by Nishimoto and Tabei [36]. OptBWTRL adds the ability to compute PLCP and ϕ in constant time with additional move data structures. It also retains a space complexity of O(r) words. We also define locally maximal exact matches (LEMs), a match that cannot be simultaneously extended in the pattern and the text instead of one that is only unable to be extended in the pattern (MEMs). Finally, we describe an algorithm for outputting all LEMs with length at least ℒ in O(m+occ) expected time given an OptBWTRL of the text and the matching statistics of the pattern with respect to the text. Note that this doesn’t result in a linear time algorithm for computing long LEMs in O(m+occ) expected time in O(r) space because an algorithm for computing matching statistics of a pattern with respect to a text in linear time in O(r) space is not known. A deterministic bound for our long LEM query algorithm is $O\left(m+occ\sqrt{\frac{\text{log}\;occ}{\text{log}\;\text{log}\;occ}}\right)$. Finally, our long LEM query admits a direct computation of long LEMs in O(mℒ+occ) expected time without being provided matching statistics as input. This algorithm may be faster than computation of matching statistics followed by O(m+occ) long LEM query in some cases, especially when ℒ is small.

It is likely that the move data structures $F\left(I_{\phi ,\text{PLCP}}\right)$ and $F\left(I_{\phi^{-1},\text{PLCP}}\right)$ can be merged into one data structure that still takes O(r) space and computes $\phi ,\phi^{-1},\text{PLCP}[i]$, and $\text{PLCP}\left[\phi^{-1}(i)\right]$ in constant time in one data structure. This would greatly reduce the number of samples needed per input interval $F\left(I_{LF}\right)$. It would also allow bidirectional movement in the SA with one input interval index. This is left as future work. Possible other future work includes a practical implementation of the structures and algorithms described here, possibly as a modification of MOVI or b-move [16, 49]. Thirdly, the ability to compute PLCP in constant time may speed up matching statistics computation in compressed space. The intuition is that when MS[i].len≤MS[i+1].len, then when MS[i].len is large, the sa-interval of P[i,i+MS[i].len-1] is small and is faster to compute with PLCP and ϕ and $\phi^{-1}$ than with reverse LF. When MS[i].len is small, computing the sa-interval is faster with reverse LF. In Heng Li's forward-backward algorithm [25], the new sa-interval is always computed by reverse LF. Computing the sa-interval with PLCP and reverse LF simultaneously is likely to be faster in practice than reverse LF alone while retaining the same worst case time complexity. The authors are currently exploring this idea. Furthermore, variable length threshold long LEMs may be useful. I.E. output long LEMs that are x% of the length of the MEMs in the same area. The authors believe a linear time algorithm for this or a similar problem given matching statistics exist. Finally, applications utilizing the long LEMs of a pattern with respect to the text is a possible fruitful direction for future work. Particularly in biological applications.

Long LEMs may have many biological applications. In general, in any application where long MEMs are used, long LEMs may also be used. Note that MEMs are a subset of LEMs and long MEMs are a subset of long LEMs. For example, in biobank scale haplotype datasets, long matches (long LEMs) in the PBWT have revealed genealogical relationships that set maximal matches (MEMs) are not able to uncover. As the compressive power of compressed string indexes increases and the number of variants in biobank scale whole genome sequencing data increases, storing unaligned genomes becomes more viable. In that case, algorithms for outputting long LEMs are needed to replace the long match algorithms in the PBWT. These matches have many applications from identity by descent segment detection, haplotype phasing, haplotype imputation, inferring genealogical relationships, and ancestry inference. Utilizing unaligned matches from a large collection of haplotype sequences instead of aligned matches from haplotypes aligned to a linear reference genome may also reduce reference bias. Finally, novel applications for long LEMs may exist, long LEMs may be used as seeds for seed and extend algorithms. They may be used as anchors for approximate matching matching algorithms [20] possibly for long read alignment to either a reference pangenome or a linear reference genome [31]. Lastly, genome to genome or genome to pangenome long LEMs detection may find similar sequences in the genomes on different genomic regions. MEMs detection may miss these similar sequences on different genomic regions because these matches will typically be overshadowed by larger encompassing matches that occur in roughly the same region in the pattern and the text. The long LEMs may therefore reveal old structural variants that MEMs and general alignment algorithms are both unable to reveal. MEMs don’t reveal these variants due to looking for only the largest matches in a region on the pattern while alignment algorithms don’t due to better alignments existing in closeby genomic regions or alignment algorithms being too computationally expensive to run on very large datasets.

Overall, we have provided a linear time algorithm for outputting all long LEMs of a pattern with respect to a text in BWT runs compressed space given the matching statistics of the pattern with respect to the text. We have also applied the move data structure of Nishimoto and Tabei to computation of PLCP in constant time. Therefore, we can compute LCP[i] given SA[i] in constant time. We apply these results to modify the OptBWTR, creating OptBWTRL. OptBWTRL is an O(r) space data structure that computes ϕ and PLCP in constant time and long LEMs in linear time given matching statistics. These algorithms result in a linear time long LEM query algorithm in uncompressed string indexes.

## A Proofs

**Lemma 3.** *The following three statements hold: (i) Let* x *be the integer satisfying $p_{x}^{+}\leq i<p_{x+1}^{+}$ for some integer* i∈[1,n]. *Then* $\phi (i)=\phi \left(p_{x}^{+}\right)+\left(i-p_{x}^{+}\right)$*; (ii)* $\phi \left(p_{\delta^{+}[1]}^{+}\right)=1$ *and* $\phi \left(p_{\delta^{+}[i]}^{+}\right)=\phi \left(p_{\delta^{+}[i-1]}^{+}\right)+d$ *where* $d=p_{\delta^{+}[i-1]+1}^{+}-p_{\delta^{+}[i-1]}^{+}$*; (iii)* $p_{1}^{+}=1$.

**Proof.** (i) Lemma 3(i) clearly holds for $i=p_{x}^{+}$. We show that Lemma 3(i) holds for $i\neq p_{x}^{+}$ (i.e., $i>p_{x}^{+}$). Let $s_{t}$ be the position in SA with sa-value $p_{x}^{+}+t$ for an integer t∈[1,y] (i.e., $SA\left[s_{t}\right]=p_{x}^{+}+t$), where $y=i-p_{x}^{+}$. Two adjacent positions $s_{t}$ and $s_{t}-1$ are contained in an interval $\left[l_{v},l_{v}+\left|L_{v}\right|-1\right]$ on SA (i.e., $s_{t},s_{t}-1\in \left[l_{v},l_{v}+\left|L_{v}\right|-1\right]$), which corresponds to the v-th run $L_{v}$ of L. This is because $s_{t}$ is not the starting position of a run, i.e. $\left(SA\left[s_{t}\right]=p_{x}^{+}+t\right)\notin \left\{p_{1}^{+},p_{2}^{+},\ldots ,p_{r}^{+}\right\}$. The LF function maps $s_{t}$ to $s_{t-1}$, where $s_{0}$ is the position with sa-value $v_{x}$. LF also maps $s_{t}-1$ to $s_{t-1}-1$, by Lemma 3(i) of [36]. The two mapping relationships established by LF produce y equalities $\phi \left(SA\left[s_{1}\right]\right)=\phi \left(SA\left[s_{0}\right]\right)+1,\phi \left(SA\left[s_{2}\right]\right)=\phi \left(SA\left[s_{1}\right]\right)+1,\ldots ,\phi \left(SA\left[s_{y}\right]\right)=\phi \left(SA\left[s_{y-1}\right]\right)+1$. The equalities lead to $\phi \left(SA\left[s_{y}\right]\right)=\phi \left(SA\left[s_{0}\right]\right)+y$, which represents $\phi (i)=\phi \left(p_{x}^{+}\right)+\left(i-p_{x}^{+}\right)$ by $SA\left[s_{y}\right]=i,SA\left[s_{0}\right]=p_{x}^{+}$, and $y=i-p_{x}^{+}$.

(ii) Let p be the integer satisfying $L_{p}=\$$. Then there exists an integer q such that $p_{q}^{+}$ is the sa-value at position $l_{p}+1\left(l_{p}+1=l_{p+1}\right)$ if p≠r; otherwise if $p=r,p_{q}^{+}$ is the sa-value at position 1 and q=1. $\phi \left(p_{q}^{+}\right)=1$, because $SA\left[l_{p}\right]=1$ always holds. Hence $\phi \left(p_{\delta^{+}[1]}^{+}\right)=1$ holds by $\delta^{+}[1]=q$.

Next, $\phi \left(p_{\delta^{+}[i]}^{+}\right)=\phi \left(p_{\delta^{+}[i-1]}^{+}\right)+d$ holds for any i∈[2,r] because (a) ϕ maps the interval $\left[p_{\delta^{+}[i]}^{+},p_{\delta^{+}[i]}^{+}+d-1\right]$ into the interval $\left[\phi \left(p_{\delta^{+}[i]}^{+}\right),\phi \left(p_{\delta^{+}[i]}^{+}\right)+d-1\right]$ by Lemma 3(i) for any i∈[1,r], (b) ϕ is a bijection from [1,n] to [1,n], and (c) $\phi \left(p_{\delta^{+}[1]}^{+}\right)<\phi \left(p_{\delta^{+}[2]}^{+}\right)<\cdots <\phi \left(p_{\delta^{+}[r]}^{+}\right)$ holds.

(iii) Recall that p is an integer satisfying $L_{p}=\$$. Then there exists an integer $q^{\prime }$ such that $p_{q^{\prime }}^{+}$ is the sa-value at position $l_{p}$. Finally, recall $SA\left[l_{p}\right]=1$. Hence, $v_{1}=v_{q^{\prime }}=1$ holds. ◀

## B Algorithm Pseudocodes

Pseudocode for the long LEM query algorithm is provided in Algorithm 1. The long LEM query algorithm is separated into subroutines to aid understanding. The long LEM query algorithm is provided as input the length threshold ℒ, the query/pattern P, and an OptBWTRL of the text T. Subroutines have access to their calling function's variables implicitly. Structures of the OptBWTRL of T are referenced directly, i.e., ND[i] instead of ${\text{OptBWTRL}}_{T}.ND[i]$. Finally, operations on move data structures use the notation of Nishimoto and Tabei [36].
